# Prediction of Residue Status to Be Protected or Not Protected From Hy-drogen Exchange Using Amino Acid Sequence Only

**DOI:** 10.2174/1874091X00802010077

**Published:** 2008-06-03

**Authors:** Nikita V Dovidchenko, Oxana V Galzitskaya

**Affiliations:** Institute of Protein Research, Russian Academy of Sciences, Institutskaya str., 4 Pushchino, Moscow Region, 142290, Russia

## Abstract

We have outlined here some structural aspects of local flexibility. Important functional properties are related to flexible segments. We try to predict regions that have been shown to exhibit the highest probability of being folded in the equilibrium intermediate or native state and will be protected from hydrogen exchange using amino acid sequence only. Our approach FoldUnfold for the prediction of unstructured regions has been applied to seven different proteins. For 80% of the residues considered in this paper we can predict correctly their status: will they be protected or not from hydrogen exchange. An additional goal of our study is to assess whether properties inferred using the bioinformatics approach are easily applicable to predict behavior of proteins in solution.

## INTRODUCTION

The functional properties of a protein molecule are a compromise between flexibility and rigidity. Structural studies have suggested the presence of loops and turns, actively fluctuating on the protein surface [1]. Important functional properties are related to these localized motions. In fact, it has been demonstrated that flexible segments are sites of immunogenicity or of proteolytic attack modulating the life span of proteins *in vivo *[2, 3]. It has been shown that regions that are more flexible than those not involved in molecular recognition.

Since flexible protein regions frequently play an important role in biological functioning, it is not surprising that the structural explanation of these dynamic properties is at present a very active area of research.

Crystallographic B factors are often used to infer the local flexibility of a folded state [4, 5]. However, comparison of many similar and identical proteins in the same and different space groups shows that the crystal packing effects generally perturb B-factor values [6]. Some loops are thought to be flexible in solution, but adopt a well-ordered beta-hairpin conformation in the crystal structure, probably only as a result of strong crystal contacts (for example, ribosomal protein s7)[7].

An ensemble of structures typically produced in nuclear magnetic resonance (NMR) refinement may exhibit structural fluctuations. A series of statistical analyses with NMR relaxation studies on proteins, whose three-dimensional structures are known, has been performed to clarify the relationship between the structural features and backbone dynamics of these proteins [8]. Comparison of average order parameters for different amino acid types indicates that amino acids with small side chains tend to have greater backbone flexibility than those with large side chains. In addition, the motions of a given NH group are also related to the sizes of the neighboring amino acids in the primary sequence [8].

Equilibrium hydrogen-exchange measurements are a powerful tool for investigating the structures, stabilities and dynamics of native and nonnative states of proteins. Information about local structural fluctuations can be obtained from the hydrogen exchange which occurs *via *local unfolding, rather than *via *global unfolding transition.

The protection factor for residue i, P_i_ = k_i_^int^/k_i_, is the ratio of the intrinsic rate, k_i_^int^, observed in an unstructured peptide [9, 10], to the observed amide hydrogen exchange rate, k_i_. When an amide hydrogen can exchange only if the protein is substantially unfolded, the local stability is equal to the global stability and the amide is said to be undergoing “global” exchange. By contrast, the so-called “local” exchange occurs through localized fluctuations of the structure and can be applied to study native state fluctuations.

Although experimental methods for measuring protection factors are well established, the detailed mechanism of the hydrogen-exchange process is not fully understood. Several theoretical works suggested algorithms to predict protection factors from protein structures.

All these methods try to find the determinants of large conformational fluctuations of proteins. One of such determinant was the accessible surface of residue area considered in the works [11]. The Gaussian network model (GNM) has been applied to the interpretation of experimental hydrogen exchange (HX) behavior of proteins in their native state or under weakly denaturing conditions [12].

Vendruscolo and the authors suggested to use a phenomenological equation to predict experimental protection factors arising from local exchange [13, 14]. The equation includes two terms which reflect the contribution of van der Waals contacts and hydrogen bonds. But all these methods use the three-dimensional structure.

Recently the method CamP (http://www-almost.ch. cam.ac.uk/camp.php) has been suggested for predicting protection factors directly from the amino acid sequence, which does not require any knowledge of the native structure of a protein [15]. The method uses a neural network. The result of predictions with such methods depends on the training dataset. The correlation coefficient is 50-70% for 12 proteins for which experimental data from hydrogen exchange have been obtained.

We suggest that the absence of protection can be explained by fluctuations of the loops between packed secondary structures. So the prediction of intrinsic disordering should identify regions of the polypeptide chain that have a tendency to undergo significant structural fluctuations. Therefore we use our program FoldUnfold [16, 17] to check our suggestion that predicted flexible regions from amino acid sequence will correlate with the status of the residue protected or not protected from hydrogen exchange in 3D structure. An additional goal of our study is to assess whether properties inferred using the bioinformatics approach are easily applicable to predict behavior of proteins in solution.

## RESULTS

### Search for Structural Parameters with the Best Correlation with  Experimental Data on Hydrogen Exchange

We try to find what structural parameter will be the best for the prediction of hydrogen exchange protection factors. We consider seven proteins for which we have experimental data: alpha-lactalbumin, equine lysozyme [18], bovine pancreatic trypsin inhibitor [19], staphylococcal nuclease [20], horse heart cytochrome c [21], staphylococcal nuclease [22] and chymotripsin inhibitor 2 (CI2) [23]. We extracted B-factor data from the corresponding PDB files from our database. We operated with two types of B-factor values. The first type of values is an average of B-factor values over all atoms of the given amino acid residue in the studied protein (). The second type of values is the B-factor value for the C_α_ atom of the given amino acid residue in the studied protein (B(C_α_)).

It turns out that the maximal correlation (47%) is obtained if to use the number of atom-atom contacts calculated from the known 3D structure, but not the B-factor (33%) and entropy scale, as the number of degrees of freedom for angles φ, ψ, and χ for each amino acid [24] (about 1%) (see Table **1**). It has been shown previously in the work of Vendruscolo [15] that the correlations between experimental protection factors and B factors are relatively weak, consistent with the view that protection factors mainly probe larger-amplitude fluctuations than B factors [25]. The CamP method gives not a very high correlation for our database of proteins (correlation coefficient is 40%). This result confirms the suggestion that the result of the program trained on the database will depend on the considered database. Vendruscolo and the authors also compared the result of disordered predictions from different programs with experimental data [15]. The authors explained weak correlations found in this case by the fact that the intrinsic propensity for being unfolded is strongly modulated by the interactions in the folded state to define the local fluctuations probed by hydrogen exchange measurements [15].

We suppose that using only amino acid sequence we can predict the status of the residue to be protected or not in 3D structure (but not absolute values) using statistics of atom-atom contacts for each 20 amino-acids sequence in the globular state (see Table **2**) [16].

### Using Fold/Unfold Method for the Prediction of Residue Status to be Protected or not from Hydrogen Exchange

In our studies, to search for status of the residue to be protected or not from hydrogen exchange using amino acid sequence only, we have employed the *FoldUnfold *program, which permits choosing different widths of the averaged window [16, 17]. As seen from our studies, the choice of the window width depends on the length of the expected loop and, consequently, on the task posed by researchers. Thus, the window width of 41 amino acid residues is optimal for the search of long unstructured regions in proteins that are considered to be completely disordered [16, 17]. The window width of 11 amino acid residues is optimal for the search of unstructured region in a polypeptide chain of 10-20 amino acid residues long.

To predict the status of the residue from the amino acid sequence, we used the scale based on the average number of contacts observed for 20 amino acids in globular proteins [16].

The procedure that was used for construction of a contact profile is as follows. First of all, contact indices are assigned to the given amino acids sequence. The second step is smoothing of the obtained profile by averaging with some sliding window. After such manipulations we translated the given body of contacts into a binary system with the following rules: if the difference between indices of the given amino acid and some threshold (the value of threshold, which is 20.4, discriminates regular and loop regions) is less than 0, it should be an unprotected amino acid. Otherwise we name this amino acid as protected. The value of the threshold and rules were taken from previous works where a contact scale was used [16, 17]. In those works it was shown experimentally that there exists correlation between contacts profile (obtained using a contact scale) and structured/unstructured regions [16, 17]. For estimation of quality of our prediction we used sensitivity and specificity. The specificity is defined as the rate of true negatives to the sum of true negatives and false positives, while sensitivity is defined as the rate of true positives to the sum of true positives and false negatives. In other words, the sensitivity defines the percentage of correct predictions of protected amino acids which are experimentally proved to be protected, while the specificity defines the percentage of correct predicted unprotected amino acids.

The FoldUnfold method with a different size of windows has been applied to seven proteins for which we have experimental data: alpha-lactalbumin, equine lysozyme [18], bovine pancreatic trypsin inhibitor [19], staphylococcal nuclease [20], horse heart cytochrome c [21], staphylococcal nuclease [22] and chymotripsin inhibitor 2 (CI2) [23].

**Fig. (1):**
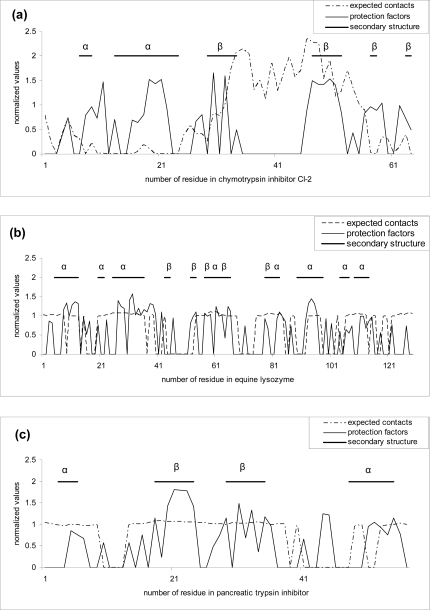
Comparison of predicted and experimental protection factors for three proteins (a) 2ci2, (b) 2eql, (c) 6pti. Dotted curves are predictions made by our FoldUnfold method with window size of 11 residues. Values that are lower than 20.4 are considered as unprotected and correspond to zero, other values normalized on the average difference between the expected number of contacts and the threshold value of 20.4. Solid curves are original protection factors presented as 0 for unprotected residues and more than 0 for protected residues (normalized on the average protection factor for protected residues). Straight lines represent regular secondary structure according to the DSSP program [26].

The results of calculations are summarized in Table **3**. As shown from the Table, the best scores belong to the size of 11 amino acids. We also tried the sliding window size of 13 amino acids, but this leads to lower results as compared to those of 11 amino acids (data not shown). One can see that for protein alpha-lactalbumin (pdb code 1hml) we have got the maximum accuracy – 97%. A little bit lower results were obtained for other proteins, thus proteins 1hrc, 2eql, 2rn2 and 6pti have about 80% of accuracy (sensitivity) on average (see Fig. **1**). The next important question that arises is whether the accuracy (sensitivity) is true in terms of specificity. We have calculated the amount of false positive predictions for every protein (specificity) as well. It can be seen from the table that the results vary greatly, from 35% to 60 %, which is about 40% on average. It should be underlined that the majority of mispredictions for proteins represent cases in which protection was predicted but not observed.

We have considered the results of predictions by the CamP method for our database of proteins to compare with our results (see Table **3**).

In spite of the fact that the CamP method is based on a neural net and gives almost 97% of accuracy, its specificity is very low (13%). There is an explanation to this fact: the method simply overpredicts the number of protected residues. According to the definition [15] the residue is protected if lnp>5, and not protected if lnp<5. Summarizing this information we can resume that in spite of its simplicity our simple approach keeps abreast with the CamP method.

## CONCLUSIONS

In this work we have shown that it is possible to predict with good accuracy (80%) the status of residues to be protected or not from hydrogen exchange directly from amino acid sequences. The optimal window size for the FoldUnfold program for prediction of the residues status to be protected or not from hydrogen exchange is 11 residues. From our analysis described in this work we can classify the loops predicted in the structure of proteins in two types. The first type includes loops in which residues are not protected from hydrogen exchange typical of the so-called flexible loops. The other type includes loops in which residues are protected and thus can be considered as rigid loops. Moreover, our results are compatible with NMR relaxation studies showing that amino acids with small side-chains tend to have greater backbone flexibility than those with large side-chains [8]. We have shown that the results of the CamP program based on a neural net depend on the considered database of proteins.

## Figures and Tables

**Table 1:** Correlation Coefficient Between Different Structural Parameters and Experimental Protection Factors

**PDB**	**B-factor (Cα)**	**Atom-atom contacts**	**CamP [[15]] **	**Entropy scale [[25]] **
1hml	0.35	-0.42	-0.43	-0.06
1hrc	0.17	-0.53	-0.54	-0.08
2ci2	0.43	-0.52	-0.10	-0.01
2eql	0.32	-0.45	-0.44	-0.10
2rn2	0.41	-0.45	-0.35	0.11
2sns	-	-0.30	-0.36	-0.10
6pti	0.31	-0.63	-0.67	-0.35
average	0.33	-0.47	-0.41	-0.08

**Table 2:** The Average Number of Contacts Per Residue in Globular State

**G**	**P**	**A**	**D**	**E**
17.1	17.4	19.9	17.4	17.5
R	H	C	V	M
21.0	21.7	23.5	23.9	24.8
K	S	N	Q	T
17.7	18.2	18.5	19.2	19.8
L	I	Y	F	W
25.4	25.7	25.9	27.2	28.5

**Table 3:** Sensitivity (fraction of correct predictions of pro-tected residues) and specificity (fraction of correct predictions of unprotected residues) characteristics of predictions made by the Fold/Unfold method with different sizes of sliding window and the CamP method

**PDB**	**11**		**9**	
	**Sensitivity**	**Specificity**	**Sensitivity**	**Specificity**
1hml	0.98	0.19	0.91	0.28
1hrc	0.79	0.68	0.68	0.65
2ci2	0.76	0.23	0.62	0.23
2eql	0.74	0.46	0.77	0.51
2rn2	0.85	0.24	0.88	0.29
2sns	0.66	0.53	0.62	0.57
6pti	0.81	0.36	0.87	0.40
average	0.80	0.38	0.76	0.42

**PDB**	**7**		**CamP**	
	**Sensitivity**	**Specificity**	**Sensitivity**	**Specificity**
1hml	0.93	0.32	1.00	0.08
1hrc	0.74	0.61	1.00	0.06
2ci2	0.68	0.23	0.91	0.27
2eql	0.70	0.53	0.99	0.08
2rn2	0.80	0.34	0.98	0.09
2sns	0.55	0.59	1.00	0.01
6pti	0.71	0.56	0.94	0.32
average	0.73	0.45	0.97	0.13
